# Supplementary material for: Ambulatory Care in Adult Congenital Heart Disease—Time for Change?
Source: J Clin Med. 2022 Apr 6;11(7):2058. doi: 10.3390/jcm11072058 (PMC9000074; doi:10.3390/jcm11072058)
Supplement: Supplementary file 1 [file jcm-11-02058-s001.zip › jcm-1650497-supplementary.pdf]

Supplementary Files

Figure S1 Ratio of decision making to holding appointments according to a) ACHD AP class and b) diagnostic category

AHA Anatomical Class	AHA Physiological Class A	B	C	D
1	0	0	0	
2	0.05	0.12	0.5	1
3		0.16	0.4	1

Diagnostic Category	
Aortic Coarctation	0.18
ASD	0
AVSD	0.45
Complex CHD	0.26
Ebstein	0.71
Fontan	0.31
Systemic RV	0.26
Tetralogy of Fallot	0.22
TGA arterial switch	0
Valvular	0.13
VSD	1

Figure S2 Number of additional investigations requested and reasons [Ix: Investigation of]

	Ix Symptoms	Ix Physical Finding	Ix ECG Finding	Ix Echo Finding	Risk Stratification	Surveillance	Treatment Planning
Bloods	8	0	0	0	2	22	1
24 Hour Blood Pressure	0	4	0	0	0	8	0
Rhythm Monitoring	22	0	6	0	0	4	0
Exercise Test	9	1	1	0	3	5	0
Advanced Cardiac Imaging	9	3	0	9	1	54	9
Cardiac Catheterisation	2	0	0	0	0	0	3
Gastrointestinal Investigations	3	0	0	0	0	10	0
Respiratory Investigations	4	0	0	0	0	0	0
Microbiology Investigation	3	0	0	0	0	0	0

Table S1 Univariate analysis of possible predictors of non-attendance [*one subject could not be categorised]

	Attender N=65	Non-Attender (≥1), N=35	p	Recurrent Non-Attender (≥2), N=15	p
Male sex	30	23	0.062	10	0.173
Median Age (Years)	40.5	39.5	0.446	40.9	0.668
ACHD A 1	5	0		0	0.405
ACHD A 2	50	26		11	
ACHD A 3	10	9	0.136	4	
ACHD P1/2	39	19		6	
ACHD P3/3	26	16	0.581	9	0.286
IMD decile 1-5*	39	21		9	
IMD decile 5-10*	25	14	0.927	6	0.996
